# Case Report: Hyper IgM Syndrome Identified by Whole Genome Sequencing in a Young Syrian Man Presenting With Atypical, Severe and Recurrent Mucosal Leishmaniasis

**DOI:** 10.3389/fimmu.2020.567856

**Published:** 2020-09-11

**Authors:** Camilla Heldbjerg Drabe, Rasmus L. Marvig, Line Borgwardt, Jens D. Lundgren, Hanne Vibeke Hansen Maquart, Terese Lea Katzenstein, Marie Helleberg

**Affiliations:** ^1^Department of Infectious Diseases, Copenhagen University Hospital, Rigshospitalet, Copenhagen, Denmark; ^2^Center for Genomic Medicine, Copenhagen University Hospital, Rigshospitalet, Copenhagen, Denmark; ^3^Department of Infectious Diseases, PERSIMUNE, Centre of Excellence for Personalised Medicine of Infectious Complications in Immune Deficiency, Copenhagen University Hospital, Rigshospitalet, Copenhagen, Denmark; ^4^Department of Clinical Immunology, Copenhagen University Hospital, Rigshospitalet, Copenhagen, Denmark

**Keywords:** immunodeficiency, leishmaniasis, hyper IgM syndrome, genetics, diagnostic, whole genome sequencing

## Abstract

A previously healthy 19-year-old Syrian man presented with atypical and severe mucosal leishmaniasis caused by *Leishmania tropica*. During a 2-year period, he had three severe relapses despite various treatment strategies, including liposomal amphotericin B and Miltefosine. Because of the unusual clinical presentation, potential underlying immunodeficiency was investigated. Normal T and NK cell counts were found. The B cell count was slightly elevated at 0.7 × 10^9^ cells/L (0.09 × 10^9^ to 0.57 × 10^9^ cells/L), but the proportions of memory and isotype switched memory B cells were severely diminished IgG levels were low, at 309 mg/dL (610–1490 mg/dL). The initial IgM and IgA levels were within normal range, but the IgA levels decreased to 57 mg/dL (70–430 mg/dL) during follow up. Common variable immunodeficiency (CVID) was initially suspected, because the immunological results of low IgG and IgA, low switched memory B cells, no profound T cell deficiency found and absence of secondary cause of hypogammaglobulinemia were compatible with this diagnosis (ESID 2019). However, the highly unusual and severe clinical presentation of *L. tropica* is not suggestive of B-cell deficiency or CVID. Eventually a pathogenic nonsense variant in the CD40 ligand gene [p.(Arg11^∗^)] was identified by whole genome sequencing, thus enabling the diagnosis of X-linked hyper IgM syndrome. This case illustrates and supports the potential for the use of whole genome sequencing in accurate diagnosis of primary immunodeficiencies.

## Introduction

Primary immunodeficiencies (PIDs) are rare diseases with heterogeneous presentations, often with symptoms appearing in early childhood. Common variable immune deficiency (CVID) is the most common clinically significant PID with an estimated prevalence of 1:50,000 to 1:25,000 (1). CVID is characterized by B cell dysfunction, which results in hypogammaglobulinemia and diminished vaccine responses. Different versions of diagnostic criteria for CVID have been used (2–4). During the past decade, the increasing availability of genetic investigation, particularly next generation sequencing, has substantially contributed to the diagnosis of PIDs. To date, more than 400 monogenic PIDs have been identified (5).

Leishmaniasis is caused by protozoan *Leishmania* parasites, transmitted by infected phlebotomine sandflies. The clinical presentations of leishmaniasis differ and include visceral, cutaneous, mucocutaneous and mucosal leishmaniasis caused by more than 20 *Leishmania* species (6). *Leishmania* are intracellular parasites, and clearance and disease control rely on a strong CD4 Th1 mediated immune response. The clinical presentation is attributed to species and host factors. Following initial infection, parasites can persist in the body in a clinically latent state. Altered immune status may lead to disease progression with clinical manifestations different from those of the original lesion (7). Immunosuppression (e.g., due to HIV or malnutrition) is associated with poor outcomes in leishmaniasis (7, 8). Underlying immunodeficiency should be considered when leishmaniasis has an atypical and/or severe presentation (9).

Here, we present the case of a young Syrian man who had three relapses of atypical and severe mucosal leishmaniasis caused by *Leishmania tropica*, thus leading to the suspicion of an underlying immune deficiency. CVID was initially suspected, because the immunological findings were compatible with the diagnostic criteria for CVID. However, the clinical presentation was not typical of CVID. Whole genome sequencing ultimately revealed a pathogenic variant in the CD40 ligand (CD40L) gene, thereby enabling diagnosis of the rare PID hyper IgM syndrome (HIGM). This case illustrates the value of genomic analyses in diagnosing PIDs.

## Case Presentation

In June 2016, a 19-year-old Syrian man with a 6-month history of throat irritation was admitted to a Danish hospital. He came to Denmark as a refugee in 2015. He had no history of severe illness or excess infections, although a scar on his arm was suggestive of previous cutaneous leishmaniasis. His family history included a mother and a sister who were healthy, and a deceased father who had also been healthy. Several days before hospital admission, the symptoms had progressed to severe throat pain and fever. Laryngoscopy revealed massive edema of the mucosa of both the pharynx and larynx. The epiglottis and uvula were swollen and red with papilloma-like lesions. A biopsy of the uvula revealed histiocytic inflammation and Donovan bodies, thus granting the diagnosis of leishmaniasis. The species *L. tropica* was identified by polymerase chain reaction. Additional paraclinical examinations included a negative HIV test, normal levels of immunoglobulin (Ig) A and IgM and low IgG (309 mg/dL). An ultrasound scan of the abdomen revealed slight splenomegaly (14 cm) and no hepatomegaly. Treatment with a standard regimen of liposomal amphotericin B (LAB) (3 mg/kg on days 1–5, 14, and 21) was initiated and led to complete resolution of the symptoms.

**FIGURE 1:**
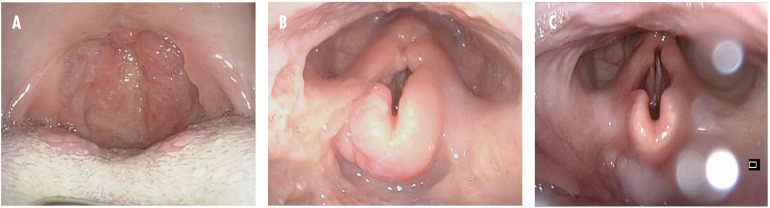
Clinical pictures from oropharyngoscopy and laryngoscopy from the third relapse. **(A)** Before treatment. The oral cavity and oropharyngeal isthmus show swelling, papilloma-like lesions, and eroded uvula. **(B)** Before treatment. Swelling of the epiglottis vestibular folds and aryepiglottic folds are seen. **(C)** One month after treatment. Near-normal findings.

However, 4 months later, the symptoms recurred. Treatment was reinitiated with LAB, but in immunodeficiency dosages (4 mg/kg on days 1–5, 10, 17, 24, 31, and 38). The IgG levels continually remained low, and immunoglobulin substitution with intravenous immunoglobulin (IVIG) was initiated. The patient showed a good clinical response, but despite ongoing immunoglobulin substitution, he was re-hospitalized in March 2017 with a second relapse. At that point, massive edema of the epiglottis threatened the airways, and tracheotomy was necessary. Miltefosine (50 mg three times daily for 28 days) was initiated, and the patient recovered completely. The patient was subsequently lost to follow-up, until he was re-admitted with a third relapse in February 2018. The uvula was completely eroded (Figure 1), and tracheotomy was again necessary because of edema. At that point, the levels of IgA and IgG were both low (57 and 220 mg/dL, respectively). The patient had responded well to all treatment regimens, and the relapses were considered likely a consequence of his immune deficiency and lack of ability to clear the intracellular parasites, rather than drug resistance. He was treated with LAB in immunodeficiency dosages, and immunoglobulin substitution was reinitiated.

After completion of the treatment for the third relapse, secondary *Leishmania* prophylaxis with Miltefosine was initiated. The initially dose was 50 mg per day, which was reduced to 50 mg three times per week, and finally to the current dose of 50 mg once weekly. The patient continues to receive immunoglobulin substitution, currently subcutaneous immunoglobulin (SCIG). As of June 2020, 2 years after the third relapse, no further relapses or other infectious episodes had occurred.

## Timeline

Figure 2.

**FIGURE 2:**
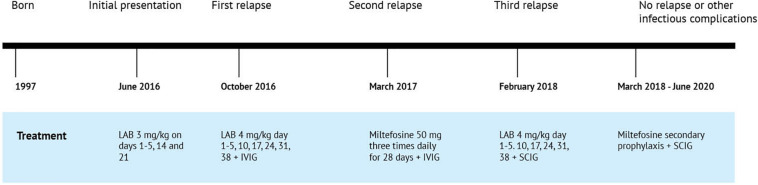
Timeline of the clinical events and treatment strategies. LAB, liposomal amphotericin B; IVIG, intravenous immunoglobulin; SCIG, subcutaneous immunoglobulin.

## Diagnostic Assessment

Because of the patient’s very unusual clinical presentation, further characterization of the immunodeficiency was conducted. The results of the complete investigation are shown in Table 1. In summary, a slightly high number of B cells were found, but a diminished proportion of memory and isotype switched memory B cells was observed. There was a severely reduced mutation fraction of somatic hypermutation of expressed immunoglobulin kappa light chain. Levels of IgG were low at the initial presentation, and by the third relapse levels of IgA were also low. Random sample of tetanus and diphtheria antibodies were measured before immunoglobulin substitution was initiated and both were low. Unfortunately, no vaccine challenge was conducted before initiation of immunoglobulin substitution. The T cell proliferation was normal, and a normal response was observed on the IL-12/IFN-γ axis.

**TABLE 1:** Results of immunological and genetic examination.

Immunologic and genetic results					
Descriptive immunologic analysis					
	Results		Reference values		Unit
**Leucocytes**	6.4		4.5–10		10^9^/L

**Lymphocytes**	1.9		0.7–4.8		10^9^/L
B cells, CD19pos	0.7		0.09–0.57		10^9^/L
T cells, CD3pos	1.2		0.69–2.70		10^9^/L
CD4pos T-cells	0.67		0.39–1.70		10^9^/L
CD8pos T-cells	0.39		0.19–1.03		10^9^/L
Ratio CD4/CD8	1.8				
NK cells, CD16/56pos:	0.09		0.08–0.56		10^9^/L

**Immune phenotype**					
**CD19pos B-cells**	37		6–18		% of lymphocytes
CD5pos B-cells	15		1–10		% of lymphocytes
CD21dim/neg B-cells	2		<2		% of lymphocytes
**B-cell subpopulations**					
Among CD19pos B-cells					
Memory B-cells (CD27pos)	4				% of cd19pos B-cells
IgM memory B-cells (CD27pos)	3		0.4–11		% of cd19pos B-cells
Isotype switched memory B-cells	<1		3–46		% of cd19pos B-cells
**CD3pos T-cells**	58		42–84		% of lymphocytes
CD4 T-cells	35		24–51		% of lymphocytes
CD8 T-cells	20		10–44		% of lymphocytes
TCR-αβ T-cells	56		35–81		% of lymphocytes
TCR-γδ T-cells	5		2–10		% of lymphocytes
HLA-DRpos TCR-αβ T-cells	12		1–6		% of TCR-αβ T-cells
HLA-DRpos TCR-γδ T-cells	1		0–1		% of TCR- γδ T-cells
**T-cell subpopulations**					
**Among CD4pos T-cells**					
Terminal differentiated CD57pos CD4 T-cells	6		0–12		% of CD4pos T-cells
CD45RAposROneg (virgin)	35		4–58		% of CD4pos T-cells
CD45RAnegROpos (memory)	56		3–59		% of CD4pos T-cells
**Among CD8pos T-cells**					
Terminal differentiated CD57pos CD8 T-cells	42		0–48		% of CD8pos T-cells
CD45RAposCCR7pos (naive)	26		20–75		% of CD8pos T-cells
CD45RAneg (memory)	56		0–38		% of CD8pos T-cells
CD45RAposCCR7neg [T-effector memory RA (TEMRA)]	18		7–53		% of CD8pos T-cells

**Immunoglobulins**					
IgA	115 (eventually dropped to 57)		70–430		mg/dL
IgG	309		610–1490		mg/dL
IgG1	140		280–800		mg/dL
IgG2	99		120–570		mg/dL
IgG3	32		24–125		mg/dL
IgG4	0.4		5.2–125		mg/dL
IgM	176		39–208		mg/dL

**Somatic hypermutation of expressed immunoglobulin kappa light chain genes**	9		27–88		Mutation fraction (%)

**Mannosebinding lektin**						<50		μg/L

**Immunologic and genetic results**					

**Functional immunologic analysis**					

	**Results**		**Reference values**		**Unit**

**Vaccine antibodies** (random samples)					
Clostridium tetani-toxin-Ab	<0.0013		>0.2		×10^3^ IU/L
Corynebacterium diphteriae-Ab	<0.00078		>0.1		×10^3^ IU/L

**Complement system**					
Classical activation	93				% of positive control
Lectin activation	0				% of positive control
Alternative activation	105				% of positive control

**Lymphocyte stimulation**					
Pokeweed Mitogen	113				% of positive control
Anti-CD3/CD28/CD2stimulation	98				% of positive control

**IFN-γ /IL-12 axis function**					
**TNF-α**	Stimuli	LPS	LPS+IFN-γ	Ratio	pg/mL
	
	Control 1	517	6697	12.95	
	Control 2	740	3100	4.19	
	
	Patient	2164	9933	4.59	
	
**IFN-γ**	Stimuli	PHA	PHA+IL-12	Ratio	
	
	Control 1	1324	6136	4.63	
	Control 2	503	7241	14.40	
	
	Patient	981	6005	6.12	
	
**IL-12p70**	Stimuli	LPS	LPS+IFN- γ	Ratio	
	
	Control 1	1	809	809	
	Control 2	1	547	547	
	
	Patient	7	3395	485	

**FIGURE 3:**
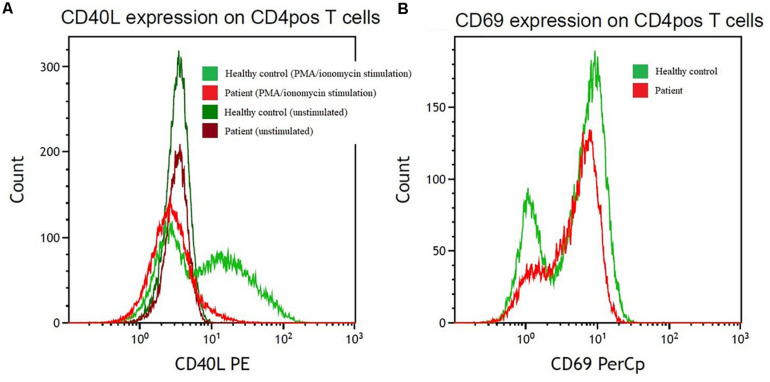
Reduction in cell surface levels of CD40L in CD4pos T cells from the index patient compared with a control individual. **(A)** CD40L expression after PMA/ionomycin stimulation (light red/light green) on CD4pos T-cells and on unstimulated (dark red/dark green) CD4pos T-cells from the index patient (red colors) compared with healthy control (green colors). **(B)** Control of PMA/ionomycin stimulation monitored by CD69 upregulation on CD4pos T cells in the patient (light red) and a healthy control (light green). PE, phycoerythrin; PerCP, peridinin chlorophyll protein complex.

**CD40L expression on activated CD3positive/CD8negative cells after stimulation with PMA/ionomycin**	Completely impaired upregulation of CD40L (Figure 3)				

**Genetic analysis**					

**Whole genome sequencing and Sanger sequencing**	A nonsense variant in CD40 ligand gene [c.31C > T, p.(Arg11*)]				

*Lymphocytes are defied by flow as CD45brigth-pos and CD14neg. CD, cluster of differentiation; NK, natural killer; Ig, immunoglobulin; Ab, antibody; IFN, interferon; IL, interleukin; TNF, tumor necrosis factor; LPS, lipopolysaccharide; PHA, phytohemagglutinin; PMA, phorbol myristate acetate; CD40L, CD40 ligand.*

Whole genome sequencing identified a pathogenic nonsense variant in the CD40L gene [c.31C>T, p.(Arg11^∗^)], which was verified by Sanger sequencing using forward and reverse strand-specific primers (5′TAACGTTTTTGCTGGGAGAGA and 5′CAAGTGCCTCAGTTTCCACAT, respectively). This variant is located on exon 1 and leads to a premature stop at codon 11. Supplementary functional analysis by flowcytometry was conducted to examine CD40L expression on activated CD4 T cells, and a marked lack of CD40L upregulation was found, thus illustrating the pathogenicity of the identified variant (Figure 3).

## Discussion

*Leishmania tropica* typically presents as cutaneous leishmaniasis with self-healing ulcerating, dry lesions (10). The atypical mucosal presentation had already prompted a suspicion of an underlying immunodeficiency at the time of initial presentation (11). The combination of the low levels of IgG and IgA, low proportion of switched memory B cells, no profound T cell deficiency and absence of secondary cause of hypogammaglobulinemia, was compatible with CVID (2). Therefore, the patient was diagnosed with CVID. However, the very unusual and severe disease presentation of *L. tropica* was not compatible with a CVID diagnosis and led us to suspect that the initial diagnosis was incorrect. The intracellular parasite infection raised suspicion of cellular deficiency, although severe manifestations of leishmaniasis have been described in patients with antibody deficiencies (12).

Genetic investigations were not part of our routine diagnostics for PID until recently, but because of emerging evidence of the benefit of genetic investigation in patients with PID (13) our patients with CVID have been offered whole exome or whole genome sequencing since 2015. In this case, the genetic investigation revealed a variant in the CD40L gene that is classified as pathogen (class five) according to ACMG guidelines (14), because it is a nonsense variant in a gene whose loss of function is a known mechanism of disease (14). The CD40L gene consists of five exons. Exon 1 encodes the intracellular, the transmembrane and a small portion of the extracellular region. Exons 2–5 encode the rest of the extracellular domain. HIGM variants typically affect the extracellular domain, thus resulting in inability of CD40-CD40L binding. Variants in exon 1 decrease the expression of CD40L (15). In this case, we found a nonsense variant in exon 1, and the pathogenicity was illustrated by impaired upregulation of CD40L on activated CD4 cells, as assessed by flowcytometry (Figure 3). The patient’s underlying immunodeficiency was therefore re-classified as HIGM rather than CVID. Further investigation into family genetics and phenotypes as well as genetic counseling of relevant family members was unfortunately impossible, because the patient is a refugee from Syria with no family in Denmark. Whether the variant is inherited or is a *de novo* variant is therefore not possible to determine.

Hyper IgM syndrome is a rare PID, which include a heterogeneous group of conditions characterized by defective immunoglobulin class-switch recombination, thus resulting in normal or elevated levels of IgM and low levels of IgG, IgA, and IgE (16). The initial symptoms indicative of HIGM often occur in early childhood, and 90% of cases are diagnosed before the age of four (17). The clinical presentation consists of recurrent and severe sino-pulmonary infections, primarily caused by encapsulated bacteria. Opportunistic infections are also frequent, e.g., Toxoplasmosis, *Pneumocystis jirovecii* pneumonia, *Cytomegalovirus* infections, chronic diarrhea, and sclerosing cholangitis in some cases caused by *Cryptosporidium* (18, 19).

The most common cause of HIGM is defects in CD40L. The inheritance is X-linked and accounts for approximately 70% of HIGM cases, which are referred to as X-linked HIGM, X-HIGM or HIGM1 (20). CD40L is expressed on activated CD4 positive T cells, and it interacts with CD40 expressed on multiple cells (such as B cells, dendritic cells, monocytes, macrophages, and platelets etc.). Binding of CD40L to CD40 on B cells provides signals for antibody production, class switching, affinity maturation and germinal center formation (21–23). Patients with HIGM typically have normal levels of B cells, but these cells are naïve, and there is a lack of switched memory B cells and deficient somatic hypermutation (17). Whereas other types of HIGM result in strictly humoral deficiencies, X-HIGM causes defective T cell priming and impaired antigen-specific T cell responses. A lack of CD40L on activated CD4 positive T cells cause less binding of CD40 on monocytes/macrophages activated by intracellular pathogens and therefore diminished Th1-cytokine responses. Thus, a lack of CD40L clinically presents as a combined immunodeficiency phenotype with high susceptibility to opportunistic infections (17, 20, 24).

Visceral leishmaniasis has been described in patients with HIGM (25, 26), but to our knowledge this is the first report of mucosal leishmaniasis leading to a HIGM diagnosis. The CD40L-CD40 pathway has been demonstrated to be central for disease control of intracellular parasites (27). The CD40L interaction with CD40 expressed on dendritic cells leads to secretion of IL-12. The failure to secrete IL-12 impairs the ability of Th1 cells to produce IFN-γ. IFN-γ is critical for activating infected macrophages, which in turn produce nitric oxide and thereby kill the *Leishmania* parasites. The importance of a strong IL-12 response in disease control of cutaneous leishmaniasis has been shown in several mouse studies, as reviewed by Okwor and Uzonna (28). In their review, the CD40L-CD40 pathway is highlighted as a central pathway for IL-12 production in both primary and secondary responses to *Leishmania*. We found normal responses when we examined the IL12/IFN-γ axis in PBMCs from our patient compared with those from healthy control individuals. This result is in agreement with findings by (29), who examined the response of the IL12/IFN-γ axis in nine X-HIGM patients and found comparable results to those in matched healthy controls. The authors speculate that the excess susceptibility to intracellular pathogens may be caused by absent CD40 activation of the effector arm of the immune system rather than by a deficient Th1 response.

Most patients with X-HIGM present in early childhood with severe infections, in contrast to our case patient, who did not report any prior excess or severe infections. However, heterogeneity in the clinical presentations of HIGM has been reported. Previous reports include patients with mild phenotypes and late presenters (15, 20, 30–32). A family of patients with the same genetic variant as that found in our case patient (p.Arg11^∗^) has been described by (30), in which the index patient presented at the age of 41 with cerebral toxoplasmosis. The patient had a brother and two nephews with the same variant. The brother had low IgG (218 mg/dL) and high IgM (475 mg/dL) levels, but no prior history of infections. The two nephews had prior histories of recurrent upper and lower respiratory tract infections. One had normal levels of IgG and IgM, whereas the other had slightly deminished IgG (420 mg/dL (540–1610 mg/dL) and slightly elevated IgM (235 mg/dL (50–180 mg/dL). Two sisters were carriers of the variant and had normal levels of IgG but mildly high levels of IgM (263 and 215 mg/dL), and no clinical signs of immunodeficiency. Another patient with the same variant (p.Arg11^∗^) has been described by (33); the patient was a previously completely healthy 8-year-old boy who presented with anemia, which was later recognized as parvovirus B19 induced anemia. The patient was treated with IVIG and hemoglobin level normalized (33).

## Conclusion

A previously healthy young man presented with a severe and recurrent *L. tropica* infection with an atypical mucosal presentation, which led to suspicion of an underlying PID. The immunological results initially led to a suspicion of CVID; however, the clinical presentation did not support this diagnosis. The rare HIGM syndrome was ultimately diagnosed through genetic investigation. This case illustrates and supports the growing potential for exome or whole genome sequencing in accurate PID diagnostics. We speculate that HIGM may be under- or misdiagnosed, and clinically milder phenotypes in particular could be misdiagnosed as other immunodeficiencies such as CVID, as occurred with our patient and has been reported previously (15). Genetic investigation is becoming increasingly available and utilized, thus potentially enabling better understanding of genotypes and phenotypes, and consequently improved care and genetic counseling for patients and family members. An improved genetic understanding might also provide new possibilities for treatment strategies. Currently, the only curative treatment for HIGM is hematopoietic stem cell transplantation, which has numerous severe limitations. In the future, modulation of the CD40L-CD40 pathway may be available as specific treatment for X-HIGM (34), and there is a potential for curative therapy with TALEN or CRISPR/Cas9 methods (35).

## Patient Perspective

The patient has expressed frustration with having a chronic illness and the need for SCIG treatment. However, he understands the necessity of the treatment to prevent severe infections. The patient has provided written informed consent for the publication of his case.

## Data Availability Statement

All datasets presented in this study are included in the article/supplementary material.

## Ethics Statement

Written informed consent was obtained from the individual for the publication of any potentially identifiable images or data included in this article.

## Author Contributions

MH, TK, JL, and CD were the clinicians in charge of patient care and management. HM oversaw immunological investigations and interpretation of results. RM and LB oversaw genetic investigations and interpretation of results. CD, MH, and TK wrote the initial manuscript draft. All authors reviewed the manuscript and contributed to the final draft.

## Conflict of Interest

TK is advisory board member or lecturer for Bristol Myers Squibb, GlaxoSmithKline, Janssen-Cilag, Merck Sharp & Dohme, Gilead, CSLBehring, Shire and Takeda; and participated in immunodeficiency conferences sponsored by CSLBehring, Shire and Takeda. CD participated in an immunodeficiency conference sponsored by Takeda. All these activities are unrelated to the present case report. The remaining authors declare that the research was conducted in the absence of any commercial or financial relationships that could be construed as a potential conflict of interest.
